# The effect of adolescent testosterone on hippocampal BDNF and TrkB mRNA expression: relationship with cell proliferation

**DOI:** 10.1186/s12868-015-0142-x

**Published:** 2015-02-18

**Authors:** Katherine M Allen, Tertia D Purves-Tyson, Samantha J Fung, Cynthia Shannon Weickert

**Affiliations:** Schizophrenia Research Institute, Sydney, Australia; Schizophrenia Research Laboratory, Neuroscience Research Australia, Barker Street, Randwick, NSW 2031 Australia; School of Psychiatry, University of New South Wales, Sydney, Australia; School of Medical Sciences, University of New South Wales, Sydney, Australia

**Keywords:** Sex steroids, Neurogenesis, Schizophrenia, Neurotrophins, Adolescence

## Abstract

**Background:**

Testosterone attenuates postnatal hippocampal neurogenesis in adolescent male rhesus macaques through altering neuronal survival. While brain-derived neurotropic factor (BDNF)/ tyrosine kinase receptor B (TrkB) are critical in regulating neuronal survival, it is not known if the molecular mechanism underlying testosterone’s action on postnatal neurogenesis involves changes in BDNF/TrkB levels. First, (1) we sought to localize the site of synthesis of the full length and truncated TrkB receptor in the neurogenic regions of the adolescent rhesus macaque hippocampus. Next, (2) we asked if gonadectomy or sex hormone replacement altered hippocampal BDNF and TrkB expression level in mammalian hippocampus (rhesus macaque and Sprague Dawley rat), and (3) if the relationship between BDNF/TrkB expression was altered depending on the sex steroid environment.

**Results:**

We find that truncated TrkB mRNA+ cells are highly abundant in the proliferative subgranular zone (SGZ) of the primate hippocampus; in addition, there are scant and scattered full length TrkB mRNA+ cells in this region. Gonadectomy or sex steroid replacement did not alter BDNF or TrkB mRNA levels in young adult male rat or rhesus macaque hippocampus. In the monkey and rat, we find a positive correlation with cell proliferation and TrkB-TK+ mRNA expression, and this positive relationship was found only when sex steroids were present.

**Conclusions:**

We suggest that testosterone does not down-regulate neurogenesis at adolescence via overall changes in BDNF or TrkB expression. However, BDNF/TrkB mRNA appears to have a greater link to cell proliferation in the presence of circulating testosterone.

## Background

Hippocampal neurogenesis may modulate hippocampal function and plasticity across life. Previous studies have suggested that estrogen and testosterone enhance neurogenesis in adult animals (reviewed in [1]). Long term gonadectomy (30+ days) in adult male rats decreases markers of hippocampal neurogenesis [2,3]. In contrast, our study in adolescent male rhesus macaques found that gonadectomy increases nacent neuron survival and increases markers of immature neurons in the hippocampus [4], suggesting that testosterone may differentially modulate neurogenesis at adolescence. Evidence from songbirds suggests that steroid hormones may regulate adult subventricular zone neurogenesis via altering synthesis of BDNF [5,6]. However, it is unknown if testosterone may trigger attenuation of neurogenesis in the adolescent mammalian hippocampus by altering levels of BDNF, a neurotrophin critically important for multiple aspects of neuronal growth, differentiation and synaptic plasticity.

The BDNF gene contains multiple 5′ untranslated exons that are spliced onto a common 3′ exon that encodes the mature BDNF protein [7-9]. Additional complexity results from alternative splice donor sites found in several of the upstream exons [7,8]. The presence of alternative 5′ promoters allows for activity-dependent, tissue specific and developmentally regulated expression of BDNF [7,10]. TrkB is found as a full length (TrkB-TK+) form that is capable of activating differentiation and survival promoting actions downstream of tyrosine kinase (TK) signalling [11] and a truncated form (TrkB-TK-) which lacks the tyrosine kinase domain in primates [12-14], or two truncated forms (TrkB-T1 and TrkB-T2) in rodents [15,16]. In rat fetal hippocampal cells, testosterone can increase BDNF expression which promotes proliferation via the full-length TrkB receptor [17]. In neurosphere cultures, BDNF signalling through truncated TrkB can promote neural stem cell proliferation [18,19].

Due to the differing action of TrkB receptors on neurogenesis, we sought to characterise the anatomical distribution of cells expressing TrkB-TK+ and TrkB-TK- within the neurogenic regions of the rhesus macaque hippocampus. We hypothesised that the degree of cell proliferation (Ki67) would be positively correlated with TrkB-TK- expression and that the degree of cell survival and/or early neuronal markers (BrdU and DCX) would be positively correlated with TrkB-TK+ expression. We also hypothesised that gonadectomy may result in increased TrkB-TK+ and BDNF expression as a mechanism to support nacent cell survival during adolescence. Increased cell survival previously identified in gonadectomised monkeys [4] may be mediated by changes in either androgen receptor (AR) or estrogen receptor (ER) signalling, as testosterone can be aromatised to estradiol. In a parallel study, we sought to determine the direct effects of adolescent testosterone on hippocampal neurogenesis (Ki67, DCX and Tuc4) and BDNF/TrkB expression by gonadectomy and replacement with testosterone, dihydrotestosterone [(DHT), a more potent androgen] and estradiol in adolescent male Sprague Dawley rats.

We found that gonadectomy or sex steroid replacement did not alter BDNF or TrkB mRNA levels in young adult male rat or rhesus macaque hippocampus. In the monkey, TrkB-TK+ mRNA showed limited expression in the neurogenic subgranular zone but was positively correlated with cell proliferation (Ki67+ cells). In the rat, Ki67 mRNA expression was positively correlated with TrkB-TK+ and BDNF mRNA expression only in the presence of sex steroids. Adolescent gonadectomy did not appear to potently influence neurogenesis in the rat.

## Methods

### Rhesus macaque gonadectomy

Primate experiments were approved by the National Institute of Health (NIH) Animal Care and Use Ethics Committee (ASP # IPC0103). All non-human primate research procedures were carried out in strict adherence to the laws and regulations of the U.S. Animal Welfare Act, (USDA, 1990) and Public Health Service Policies, (PHS, 2002) as well as non-governmental recommendations of the National Research Council as published in the ILAR “Guide for the Care and Use of Laboratory Animals”. All research facilities were approved by the International Association for the Assessment and Accreditation of Laboratory Animal Care”. A total of twelve experimentally naive male rhesus macaque monkeys (*Macaca mulatta)* from the NIH Animal Center’s primate field station were recruited into this study. Subjects, housing conditions, surgery and endocrine measurements for this cohort have been described in detail in Richards et al. [20]. Briefly, at 29 months of age (~2.4 years, prior to adolescent testosterone increase) monkeys were either gonadectomised (Gdx, *n* = six) or sham-operated (intact, *n* = six) with a monkey from each group undergoing a yoked surgery (simultaneous anaesthesia, timing and recovery). Plasma testosterone levels were measured every 6–8 weeks beginning 1 month prior to surgery and continuing to the conclusion of the study [20,21].

Concentrations of circulating testosterone, estradiol and luteinising hormone indicated that gonadectomy was complete. Both the average evening (measured between 38 months of age and sacrifice) and the last evening (before sacrifice) testosterone levels were almost 100 times higher in the intact group (14.29, 13.19 ng/ml, respectively) compared to the Gdx group (0.15, 0.09 ng mL − 1, respectively) (*t*(5.0) > 7.35, *p* < 0.0007). Fourteen days prior to sacrifice all subjects received BrdU injections (50 mg/kg i.p. in a 20 mg/kg saline with 0.01 M NaOH) into the right side of the abdomen. A total of four injections were performed in a single day for each animal, spaced 3 hr apart [4]. At 55 months of age, animals were deeply anaesthetised with ketamine (10 mg/kg i.m.), xylazine (2 mg/kg i.m.), and pentobarbital (35 mg/kg i.p.) and perfused with 0.9% saline with 0.05% heparin. Brains were removed, hemisected and cut into 1 cm coronal blocks. Blocks were frozen in −40°C isopentane then stored at −80°C. Series of thick and thin coronal sections were cut from frozen left hemisphere hippocampal blocks. 14 μm sections were thaw mounted on glass slides. 60 μm sections were collected in wax paper and the hippocampal formation (including the parasubiculum and fimbria) was micro dissected over dry ice with chilled feather weight scissors.

### Rat gonadectomy and sex steroid replacement

The experiments conducted with rats were approved by the Animal Care and Ethics Committee of the University of New South Wales in accordance with the National Health and Medical Research Council of Australia’s Code of Practice for the Care and Use of Animals for Experimental Purposes, which also conforms to standard international guidelines (Ethics number ACEC10/40). Male Sprague Dawley rats were used for all experiments (Animal Resource Centre, Perth, WA, Australia). Rats were group housed (3-4/cage) in 12/12 hr light/dark phases with constant humidity and temperature and free access to water and standard rat chow. Subjects, housing conditions, surgery and endocrine measurements for this cohort have been described in detail in Purves-Tyson et al. [22]. Briefly, adolescent male rats were gonadectomised at 45 days of age, prior to the adolescent testosterone increase, and immediately given continuous replacement testosterone (T), dihydrotestosterone (DHT) or 17β-estradiol (estradiol, E) by subdermal silastic implant [23-25] for two weeks. By 60 days of age, intact animals have experienced the increase in testosterone associated with adolescence [26,27]. Rats were divided into five experimental groups: intact (Intact, n = 15), gonadectomy alone (Gdx, n = 14); gonadectomy plus testosterone (Gdx + T, n = 15); gonadectomy plus DHT (Gdx + DHT, n = 14), gonadectomy plus estradiol (Gdx + E, n = 15). The intact animals underwent sham abdominal surgery but gonads were left in place. Rats that did not receive hormone replacement (Intact and Gdx) received a blank implant.

Serum testosterone levels and seminal vesicle weights were used to confirm successful gonadectomy, and have been described in Purves-Tyson et al. [22]. Average circulating serum testosterone was 0.03 ± 0.001 ng/ml in the Gdx group (n = 9), 2.8 ± 0.6 ng/ml (n = 12) in the Intact group and 23.1 ± 12.0 ng/ml in the Gdx + T group (n = 14). Average circulating serum DHT was not detectable in the Gdx group, 0.2 ± 0.03 ng/ml in the Intact group, and 21.42 ± 10.6 ng/ml in the Gdx + DHT group. Circulating serum estradiol was 7.3 ± 0.15 pg/ml in the Intact group and 17.0 ± 6.7 pg/ml in the Gdx + E group. Gdx reduced seminal vesicles to 9.0% weight of Intact group. Seminal vesicles were maintained at 94.2% of intact weights by replacement testosterone and at 44% of intact weights by replacement DHT. Estradiol replacement had no effect on seminal vesicle weights such that the Gdx + E group was not significantly different to the Gdx group. At 60 days of age rats were anaesthetized with 60 mg/kg sodium pentobarbital (Euthal, Delvet, Seven Hills, Australia). Brains were removed from the skull and the hippocampus was dissected whole by severing the fimbria/fornix and flipping the hippocampus away from the cortex, following the Rat Brain Atlas as a guide [28].

### RNA extraction and cDNA synthesis

Primate RNA extraction was performed on 8 × 60 μm left hemisphere hippocampal sections, and cDNA synthesis was performed on 2 μg as previously described [4]. In the rat, RNA was extracted from randomly selected left or right hemisphere hippocampus as before [22]. cDNA synthesis was performed on 2 × 3 μg total RNA as described previously [22].

### Real time quantitative PCR

RT-PCR was performed in triplicate, by TaqMan Gene Expression Assays (Applied Biosystems, Foster City, CA, USA) using an ABI Prism 7900HT Fast Real-Time PCR System and a 384-well format. In rodents, the geometric mean of the expression of two housekeeping genes (YWHAZ and GUSB) was used to calculate the normalizing factor for gene expression, and were selected on the basis that they did not differ between treatment conditions [*F*_4,64_ = 0.62, *p* = 0.65]. Samples were run alongside a seven point standard curve using hippocampal cDNA derived from a subset of 20 cases, including samples from all groups. In monkeys, TBP and SDHA were used as housekeeping genes, on the basis that their geometric mean was unchanged by gonadectomy [*t*_10_ = 0.96, *p* = 0.36)]. Samples were run alongside a seven point standard curve using serial dilutions of cDNA derived from hippocampus RNA pooled from all 12 monkeys. Table 1 shows the transcripts that were measured in monkeys and rats, which were selected in part on the basis of being among the most highly expressed BDNF transcripts in the human and rat hippocampus [7,8].

**Table 1:** **RT-qPCR assays**

**Gene**	**Monkey**	**Rat**
TrkB-TK+	Hs0193098_m1	Rn00820626_m1
TrkB-TK-	Hs0193110_m1	
TrkB-T1		AJSO7AX
TrkB-T2		AJRR84P
BDNF I	Hs00538277_m1	Rn01484924_m1
BDNF II	Hs00538278_m1	
BDNF IIA		Rn00560868_m1
BDNF IIC		Rn01484925_m1
BDNF III		Rn04230563_m1
BDNF IV		Rn01484927_m1
BDNF VI	Hs00601650_m1	Rn01484928_m1
MIK67		Rn01451446_m1
DCX		Rn00670392_m1
DPYSL3		Rn00564016_m1
SDHA	Hs01549169_m1	
TBP	Hs0042760_m1	
YWHAZ		Rn00755072_m1
GUSB		Rn00566655_m1

### In situ hybridisation

Specific riboprobes were generated from human TrkB-TK+ and TrkB-TK- containing plasmids as previously described [13]. Antisense riboprobes and sense strand RNAs were generated from linearized plasmid using SP6 and T7 polymerases respectively and an *in vitro* transcription kit as recommended by the manufacturer (Promega, WI, USA). [^35^S] antisense and sense riboprobes were labelled to a specific activity of ~2 × 10^9^ cpm/μg by addition of radiolabelled UTP and were purified by ethanol precipitation. *In situ* hybridisation histochemistry was performed on 14-μm coronal sections containing the hippocampus as previously described [29]. Slides were exposed to film for one week or dipped in photographic emulsion (Kodak, type II NTB) for 12 weeks and then developed and counterstained with thionine. All sections were assayed together to limit interassay variability.

### In situ film analysis

Calibrated densitometric image analysis (ImageJ, National Institutes of Health) was conducted blind to treatment group on all autoradiographs (2 slides/case). Three randomly placed lines were drawn perpendicular to the dentate gyrus, and the average peak optical density was calculated for each case. The area of the hilus was outlined according to the Rhesus Macaque Brain Atlas [30], and mean optical density of this region was measured. The density of TrkB mRNA (in μCi/g) was computed from optical density data using radioactive standards (American Radiolabeled Chemicals Inc., MO, USA).

### Silver grain analysis

Slides were examined using a Nikon Eclipse 80i light microscope, and the virtual slice facility on StereoInvestigator (MBF Biosciences, Wilson, VT) was used to generate images of the region including the dentate gyrus. Using ImageJ (NIH, Maryland) 80 μm × 80 μm boxes were semi-randomly placed, with the top of the box aligned with the lower edge of the granule cell layer. 10 boxes were placed per slide. Cells positive for TrkB mRNAs (defined as a cluster of silver grains concentrated over a counter-stained nuclei) were counted. The location of positive cells within each counting box was noted, and separate counts were made for cells located within the upper 40 μm of the box, closest to the granule cell layer [i.e. subgranular zone [31]], or in the lower 40 μm of the box (i.e. hilus). The average density of labelled cells in each region for each subject was calculated.

### Ki67 and BrdU Immunohistochemistry

Immunohistochemistry for Ki67 and BrdU and cell density measurements were previously performed in Allen et al. [4]. We have briefly re-described the methods used for the sake of clarity. Standard diaminobenzidine protocols were performed on fresh frozen 14 μm sections to detect Ki67 [Abcam, Cambridge, UK, cat# ab15580, [32]] and BrdU [BD Biosciences, Franklin Lakes, NJ, cat# 347580, [33]]. BrdU assays included antigen retrieval with 2 M HCl at 37°C for 30 mins. Control slides were incubated with secondary antibody without primary antibody for each assay. Cell counts were performed in the dentate gyrus/subgranular zone (using Nikon Eclipse 80i light microscope). The cross-sectional area of the dentate gyrus was measured using StereoInvestigator (MBF Biosciences, Wilson, VT).The density of each marker (cells/mm^2^) was calculated and averaged across ~16 slides/monkey spaced evenly across the extent of the dentate gyrus.

### Statistical analysis

Outliers were removed by Grubb’s test (GraphPad QuickCalcs online calculator). This resulted in the removal of 2 data points from the rhesus macaque data (1.04% of all data) and 5 data points from the Sprague Dawley data (0.6% of all data). Two-tailed paired sample t-test were performed to analyse regional differences in TrkB mRNA+ cell localisation in monkeys. Two-tailed independent sample t-tests were performed to analyse the difference in mRNA expression between intact and gonadectomised monkeys, data are presented as percent change in mRNA levels relative to the intact group ± SEM. One-way ANOVA was performed to determine differences in mRNA expression between intact, gonadectomised and sex hormone replaced rat groups, data are presented as percent change in mRNA levels relative to the intact group. Pearson’s correlations were performed to analyse the relationship between neurogenic markers (Ki67, DCX and TUC4) and neurotrophin expression. In rhesus macaque, planned comparisons were between TrkB variant mRNA expression and measures of neurogenesis (Ki67+ cells/mm^2^, BrdU+ cells/mm^2^ and DCX mRNA expression). In the rat, planned comparisons were between TrkB variant mRNA expressions and measures of neurogenesis (Ki67, DCX and TUC4 mRNA expression) in all treatment groups combined. Pearson’s correlations were also used to analyse the relationship between Ki67 mRNA expression and BDNF or TrkB variant mRNA expression in each rat treatment group separately. All statistical tests were performed with STATISTICA 12 (StatSoft; Tulsa, OK) and all graphs were produced with GraphPad Prism 6 (GraphPad Software, La Jolla, CA, RRID: rid 000081). Significance was set at *p* < 0.05 for all tests.

## Results

### TrkB-TK+ and TrkB-TK- mRNA expression in hippocampal subregions in the monkey

**Figure 1:**
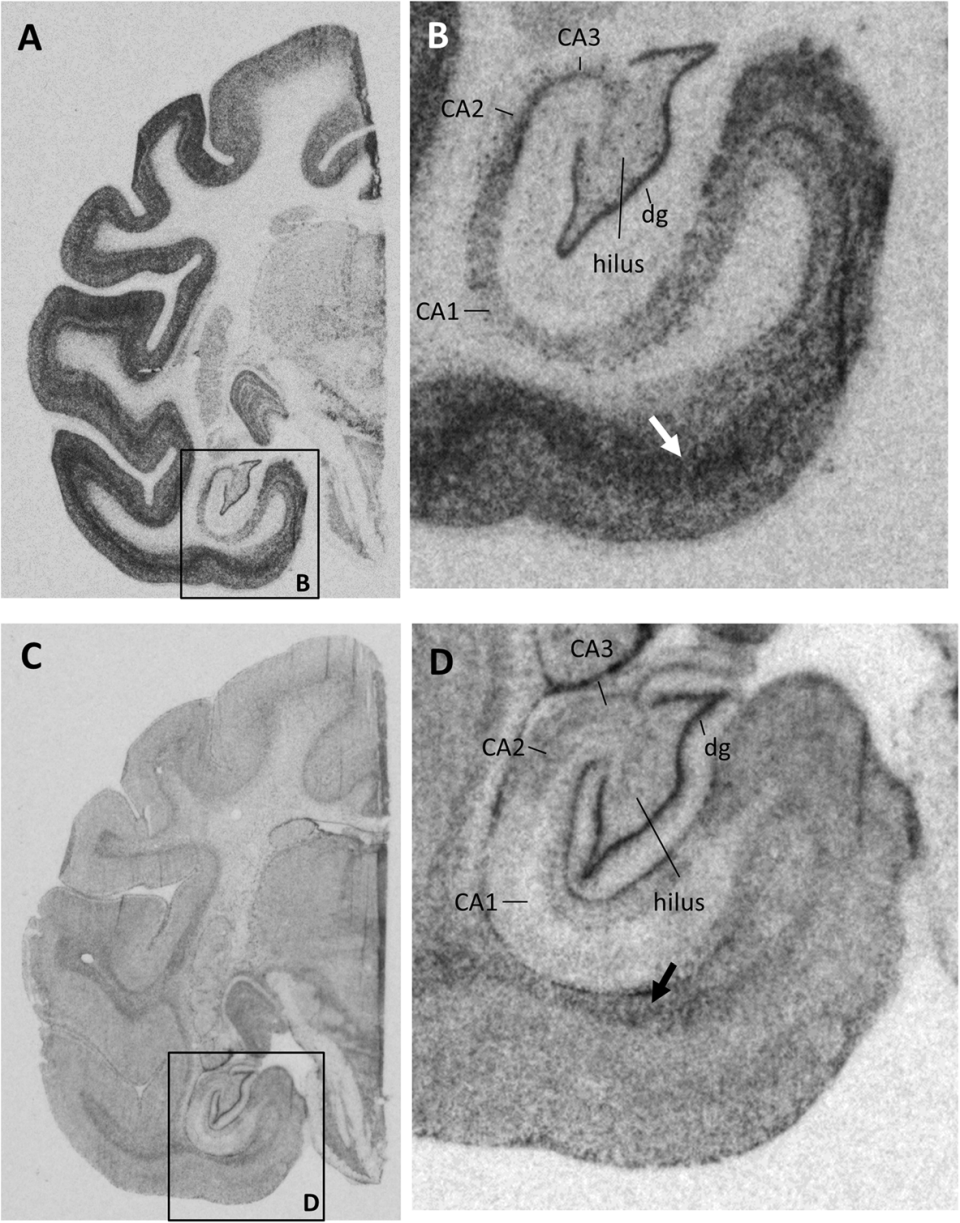
**Representative autoradiographs of TrkB mRNA in situ hybridisation in coronal sections of the rhesus macaque hippocampus. (A-B)** TrkB-TK+ mRNA was particularly highly expressed in the dentate gyrus, CA2 and the middle layers of the parahippocampal cortex (white arrow). **(C-D)** TrkB-TK- mRNA was highly expressed in the dentate gyrus and moderately expressed in the hilus and entorhinal cortex, but was much lighter in the CA1 region. TrkB-TK- mRNA was more highly expressed in the superficial white matter (black arrow). dg = dentate gyrus.

**Figure 2:**
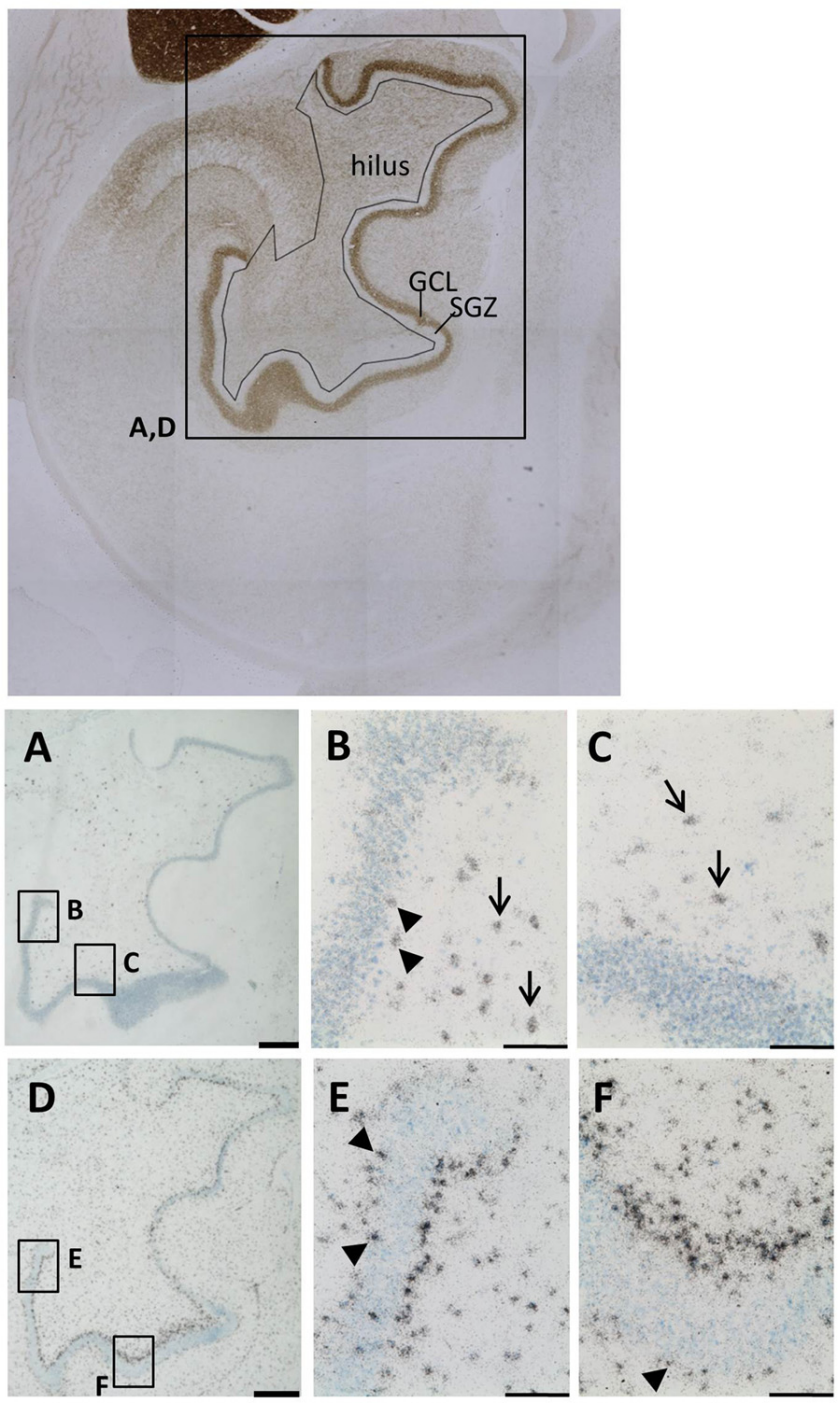
**Representative photomicrograph of silver grains on TrkB mRNA+ cells in the rhesus macaque hippocampus.** Top panel: Acetylcholinesterase stained coronal section of the hippocampal formation. The dark brown band of tightly packed cells is the granule cell layer of the dentate gyrus (GCL). The subgranular zone (SGZ) is the ~40 μm wide band underneath the GCL. The approximate location of the boundary between the SGZ and hilus is indicated with a black line. **(A)** Localisation of TrkB-TK+ mRNA+ cells. Scale bar = 500 μm. **(B-C)** The majority of TrkB-TK+ mRNA+ cells were found more than 40 μm beneath the dentate gyrus into the hilus (arrows). A limited number of TrkB-TK+ mRNA+ cells were found within the subgranular zone (arrowheads). Scale bar = 100 μm. **(D)** Localisation of TrkB-TK- mRNA+ cells. Scale bar = 500 μm. **(E-F)** TrkB-TK- mRNA+ cells are concentrated within the subgranular zone (arrows). A limited number of cells on the outer edge of the granule cell layer are also TrkB-TK- mRNA + (arrow heads). Scale bar = 100 μm.

As distinct TrkB isoforms appear to regulate different stages of neurogenesis [19], we investigated the presence of TrkB-TK+ and TrkB-TK- mRNA in the subgranular zone, granule cell layer and hilus in the rhesus macaque. Film-based analysis indicated that mRNA encoding the two TrkB receptors showed differential anatomical expression patterns in the brain in general and in the hippocampal subregions more specifically. TrkB-TK+ mRNA (Figure 1 A-B) showed widespread expression in grey matter areas including the hippocampal formation, and was particularly highly expressed in the dentate gyrus, CA2 and the middle layers of the parahippocampal cortex (arrow Figure 1B). In contrast, TrkB-TK- mRNA (Figure 1C-D) was found at relatively low levels of expression in the grey matter and appeared to be higher in superficial white matter areas, with an apparent band of increasing intensity at the grey matter /white matter border corresponding to superficial white matter (arrow Figure 1D, more clear laterally and dorsally, Figure 1C). Like TrkB-TK+ mRNA, TrkB-TK- mRNA also showed high expression in the dentate gyrus and moderate expression in the hilus and entorhinal cortex, but TrkB-TK- was largely absent from the CA1 region. Microscopic inspection of silver grain positive cells indicated that TrkB-TK+ mRNA (Figure 2A) shows moderate expression in cells within the subgranular neurogenic zone (arrow heads, Figure 2B) and an even distribution across the granule cell layer and high expression within some large cells within the hilus region (arrows, Figure 2B-C). However, TrkB-TK- mRNA positive cells were particularly concentrated just under the granule cell layer along the subgranular zone (Figure 2D-F). In contrast to TrkB-TK+, TrkB-TK- mRNA showed limited expression within the granule cell layer itself, although there were numerous individual cells at the boundary of the granule cell and molecular layers which expressed TrkB-TK- but these TK- expressing cells found superficial to the granule cell layer did not appear as densely packed as the TK-expressing cells found deep to the granule cell layer (arrow heads, Figure 2E-F). There was an average of 90 TrkB-TK+ mRNA+ cells/mm^2^ in the subgranular zone (first 40 μm from granule cell layer) and an average of 122 TrkB-TK+ mRNA+ cells/mm^2^ in the outer 40 μm. There was an average of 1102 TrkB-TK- mRNA+ cells/mm^2^ in the subgranular zone, and an average of 344 TrkB-TK- mRNA+ cells/mm^2^ in the hilus. There was a significantly higher density of TrkB-TK- mRNA+ cells in the subgranular zone compared to TrkB-TK+ mRNA+ cells [*t*_11_ = 16.02, *p* < 0.0001].

### Adolescent gonadectomy does not alter hippocampal BDNF or TrkB mRNA expression in the monkey

**Figure 3:**
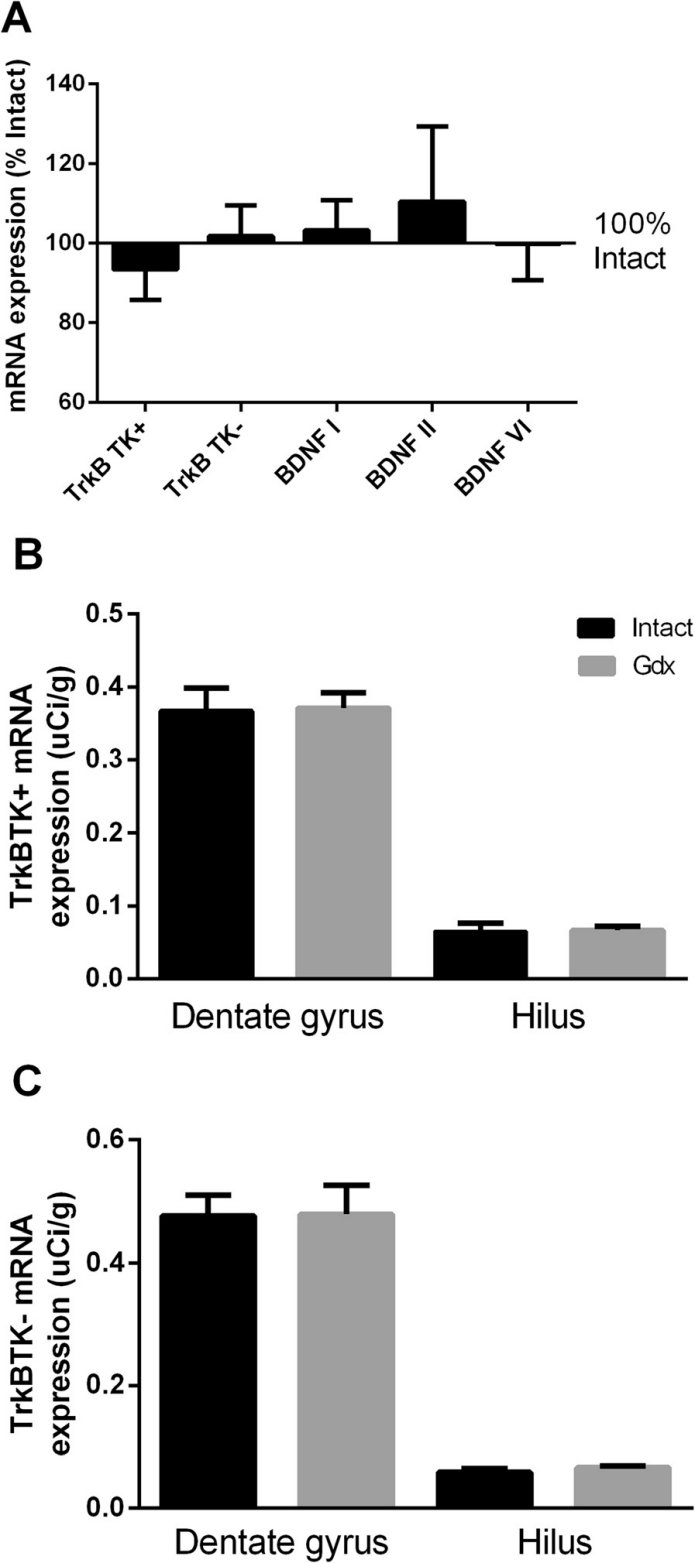
**Hippocampal expression of BDNF and TrkB mRNAs in rhesus macaque. (A)** Gonadectomised monkeys did not have significantly different expression of BDNF or TrkB mRNAs compared to intact monkeys (100%) by qPCR (all *p* > 0.05). Graph shows gonadectomised group mRNA expression (mean ± SEM) as % of intact group expression. Gonadectomy did not alter the expression of **(B)** TrkB-TK+ mRNA or **(C)** TrkB-TK- mRNA in the dentate gyrus or hilus by *in situ* hybridisation (all *p* > 0.05) in intacts (black bars) and Gdx (grey bars).

In the monkey, there were no significant group differences in mRNA expression of any BDNF or TrkB transcript examined (see Table 1) between Gdx and Intact animals as measured by qPCR (Figure 3A; all *p* > 0.05). Further, we did not detect a change between intact and gonadectomised animals in TrkB-TK+ or TrkB-TK- mRNA in the dentate gyrus or hilus by *in situ* hybridisation (film-based) (both *p* > 0.05, Figure 3B,C). Analysis of silver grain positive cells indicated that gonadectomy did not alter the density of TrkB-TK+ or TrkB-TK- mRNA+ cells in the hilus or subgranular zone (all *p* > 0.05) (data not shown).

### TrkB-TK+ mRNA is correlated with Ki67 density in the monkey

**Figure 4:**
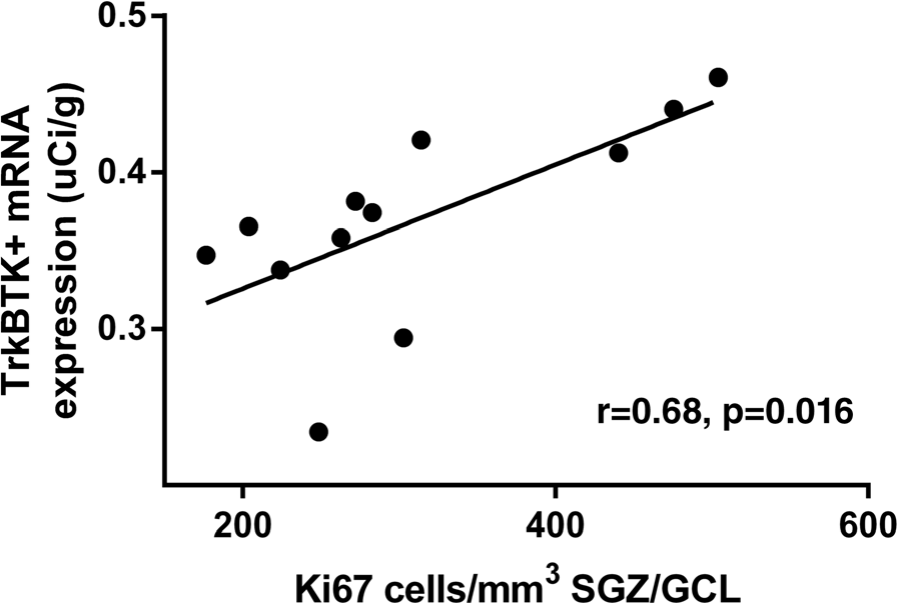
**TrkB-TK+ mRNA expression (film-based) in the dentate gyrus was positively correlated with the density of Ki67 labelled cells in rhesus macaque subgranular zone + granule cell layer.**

We hypothesised that TrkB-TK- mRNA expression in the dentate gyrus would be related to our previously calculated measure of cell division (Ki67+ cells/mm^3^) in this cohort [4]. Contrary to our hypothesis, we found a positive correlation between dentate gyrus TrkB-TK+ mRNA expression (*in situ* hybridisation) and Ki67+ cells/mm^3^ in the dentate gyrus (*r*_10_ = 0.68, *p* = 0.016) (Figure 4). Conversely, the truncated forms of the receptor, which we expected to correlate with our proliferation marker, showed no relationship with Ki67 cells/mm^3^ in monkey (Table 2). Further, we failed to detect a strong relationship between TrkB-TK+ mRNA levels and our index of maturing neuroblasts [DCX (*r*_10_ = 0.42, *p* = 0.20)]. Neither form of the TrkB receptor mRNA was correlated with cell survival (BrdU+ cells/mm^3^) in the dentate gyrus (all *p* > 0.05).

**Table 2:** **Correlations between TrkB mRNA expression and neurogenic markers in the hippocampus**

**All monkeys**	**r-value**	**p-value**
Ki67 vs TrkB-TK+	**0.68**	0.016*
Ki67 vs TrkB-TK-	−0.19	0.56
DCX vs TrkB-TK+	0.40	0.20
DCX vs TrkB-TK-	−0.035	0.91
BrdU vs TrkB-TK+	0.42	0.20
BrdU vs TrkB-TK-	0.18	0.60
**All rat groups**	**r-value**	**p-value**
Ki67 vs TrkB-TK+	**0.80**	<0.00001***
Ki67 vs TrkB-T1	0.19	0.12
Ki67 vs TrkB-T2	0.021	0.86
DCX vs TrkB-TK+	**0.88**	<0.00001***
DCX vs TrkB-T1	0.064	0.60
DCX vs TrkB-T2	0.077	0.53
TUC4 vs TrkB-TK+	**0.88**	<0.00001***
TUC4 vs TrkB-T1	0.23	0.058
TUC4 vs TrkB-T2	0.16	0.21

In all 12 rhesus macaques, TrkB-TK+ mRNA expression (*in situ* hybridisation) was positively correlated with the density of Ki67 labelled cells in the dentate gyrus. In all Sprague Dawley rat treatment groups, TrkB-TK+ mRNA expression was positively correlated with Ki67 mRNA expression, DCX mRNA expression and TUC4 mRNA expression. *p < 0.05, ***p < 0.001, bold type=p≤0.05.

### Adolescent testosterone replacement may decrease cell proliferation but does not alter markers of immature neurons in the rat hippocampus

**Figure 5:**
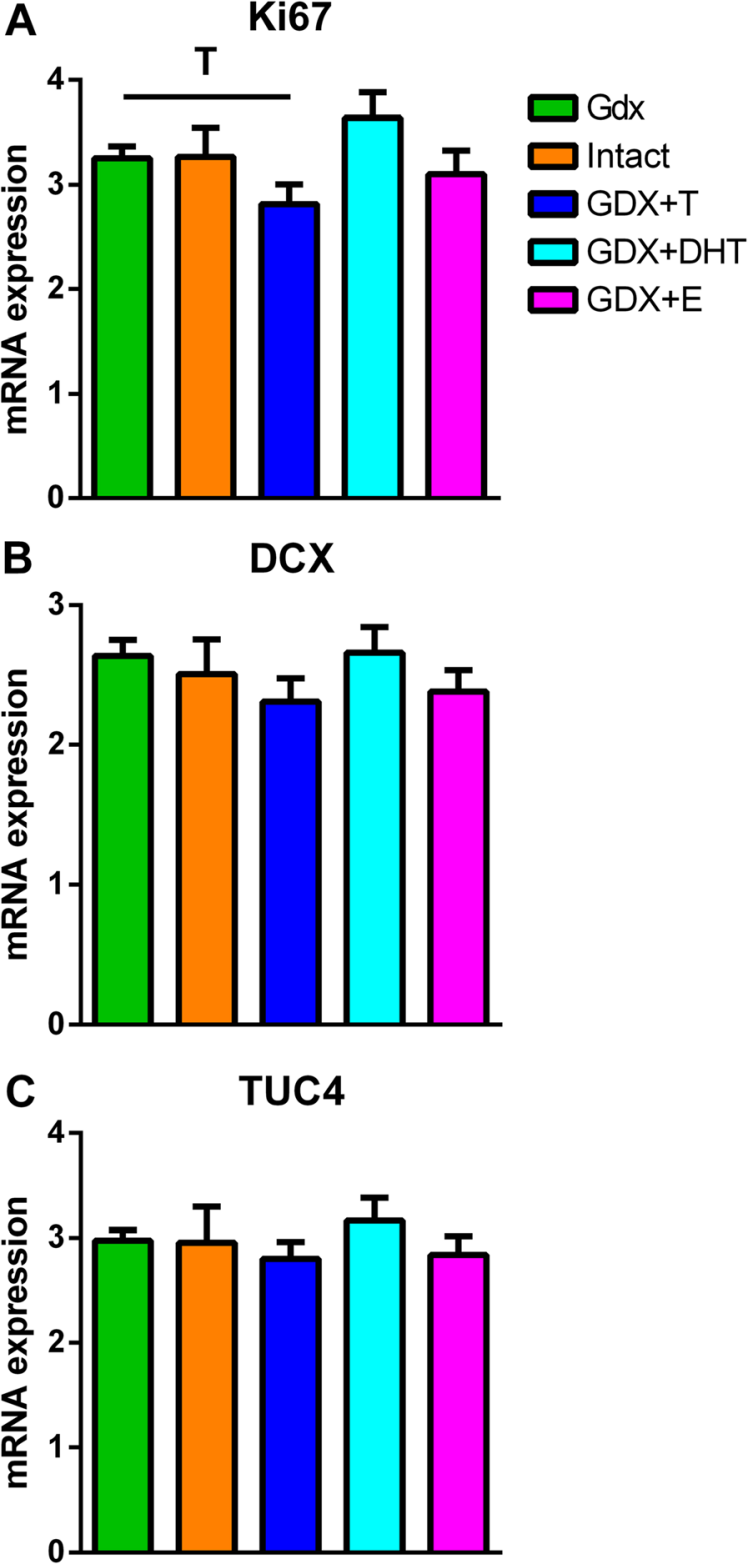
**Hippocampal expression of neurogenic markers in Sprague Dawley rats. (A)** Hippocampal expression of Ki67 (MKI67) mRNA showed a trend to be decreased in the GDX + T group compared to the GDX group (^T^ *p* = 0.055). **(B)** Doublecortin (DCX) and **(C)** TUC4 (DPYSL3) mRNAs were not significantly different between treatment groups (both p > 0.05). Graphs show mean ± SEM. Gdx = gonadectomised, Gdx + T = gonadectomised + testosterone replacement, Gdx + DHT = gonadectomised + dihydrotestosterone replacement, Gdx + E = gonadectomised + estradiol replacement.

In Sprague Dawley rats there was no overall significant treatment effect on hippocampal mRNA expression of Ki67 [(MKI67) (*F*_4.64_ = 1.8, *p* = 0.13)], doublecortin [(DCX) (*F*_4,64_ = 0.76, *p* = 0.56)] or TUC4 [(DPYSL3) (*F*_4, 63_ = 0.45, *p* = 0.77)] (Figure 5). Rats with two weeks of testosterone replacement (Gdx + T) did show a trend towards a decrease in Ki67 mRNA expression (*t*_25_ = 2.01, *p* = 0.055) compared to Gdx rats that received no hormone replacement.

### Adolescent gonadectomy or sex hormone replacement does not alter hippocampal BDNF or TrkB mRNA expression in the rat

**Figure 6:**
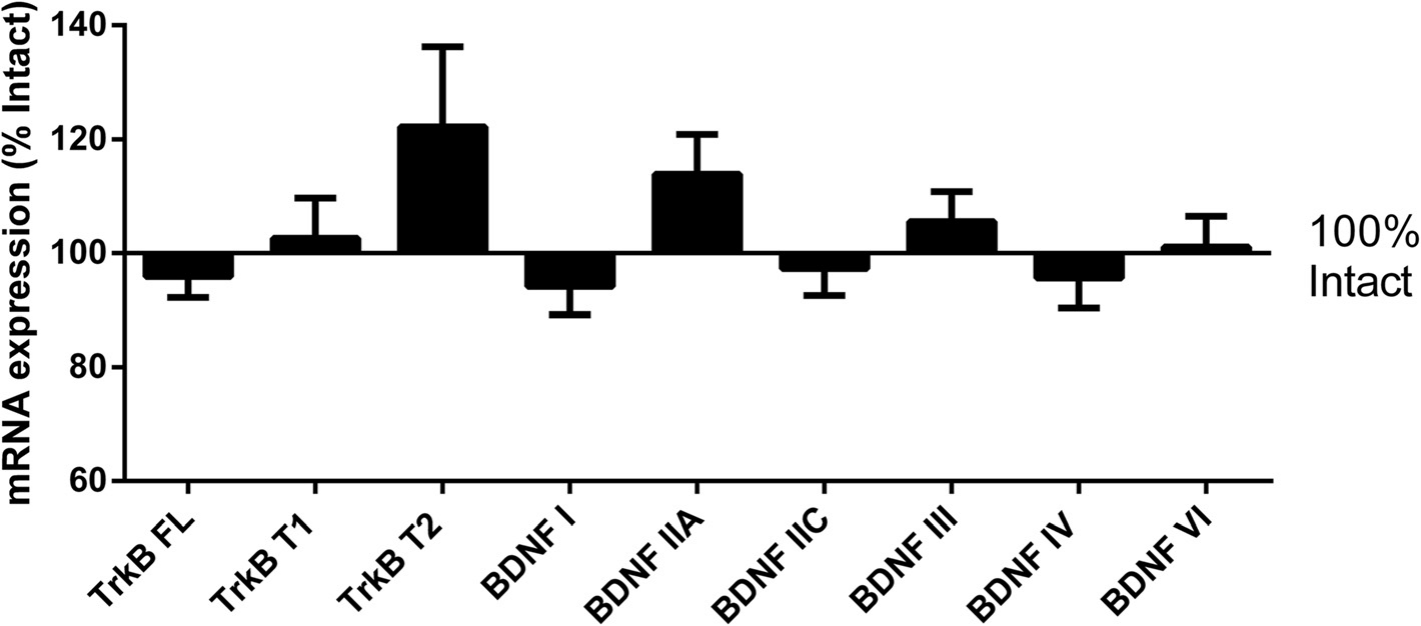
**Hippocampal expression of BDNF and TrkB mRNAs in Sprague Dawley rats.** Gonadectomised rats did not have significantly different expression of BDNF or TrkB mRNAs compared to intact rats (all *p* > 0.05). Graph shows gonadectomised group mRNA expression (mean ± SEM) as % of intact group expression.

Similar to our findings in monkeys, gonadectomy did not alter the expression of any BDNF or TrkB transcript (Figure 6) relative to intact in the rodent hippocampus. In addition, sex hormone replacement (testosterone, DHT or estradiol) did not alter expression of any transcript (one way ANOVA, all *p* > 0.05, results not shown).

### TrkB-TK+ is correlated with Ki67 mRNA expression in the rat

**Figure 7:**
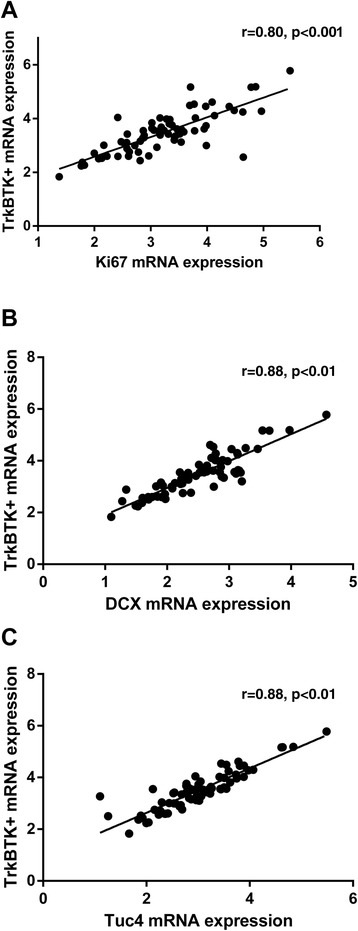
**Correlations between TrkB mRNA expression and neurogenic markers in the hippocampus.** In Sprague Dawley rats, TrkB-TK+ mRNA expression was positively correlated with **(A)** Ki67 mRNA expression, **(B)** DCX mRNA expression and **(C)** TUC4 mRNA expression.

We replicated the unexpected positive correlation between TrkB-TK+ receptor and Ki67 mRNA expression we found in the monkey, in the rat (*r*_66_ = 0.80, *p* < 0.00001) (Table 2; Figure 7A). Furthermore, we confirmed that the mRNA for the truncated forms of TrkB, TrkB-T1 and TrkB-T2, showed no relationship with Ki67 mRNA levels in the rodent hippocampus contrary to our original hypothesis but in line with our results from the male monkey hippocampus (all *p* > 0.05) (Table 2). Consistent with our original hypothesis, full length TrkB showed a positive correlation with DCX (*r*_66_ = 0.88, *p* < 0.00001) (Figure 7B) and TUC4 (*r*_66_ = 0.9104, *p* < 0.001) (Figure 7C) in the rat hippocampus.

### Circulating sex hormones alter the relationship between cell proliferation and BDNF and TrkB expression in the rat

**Figure 8:**
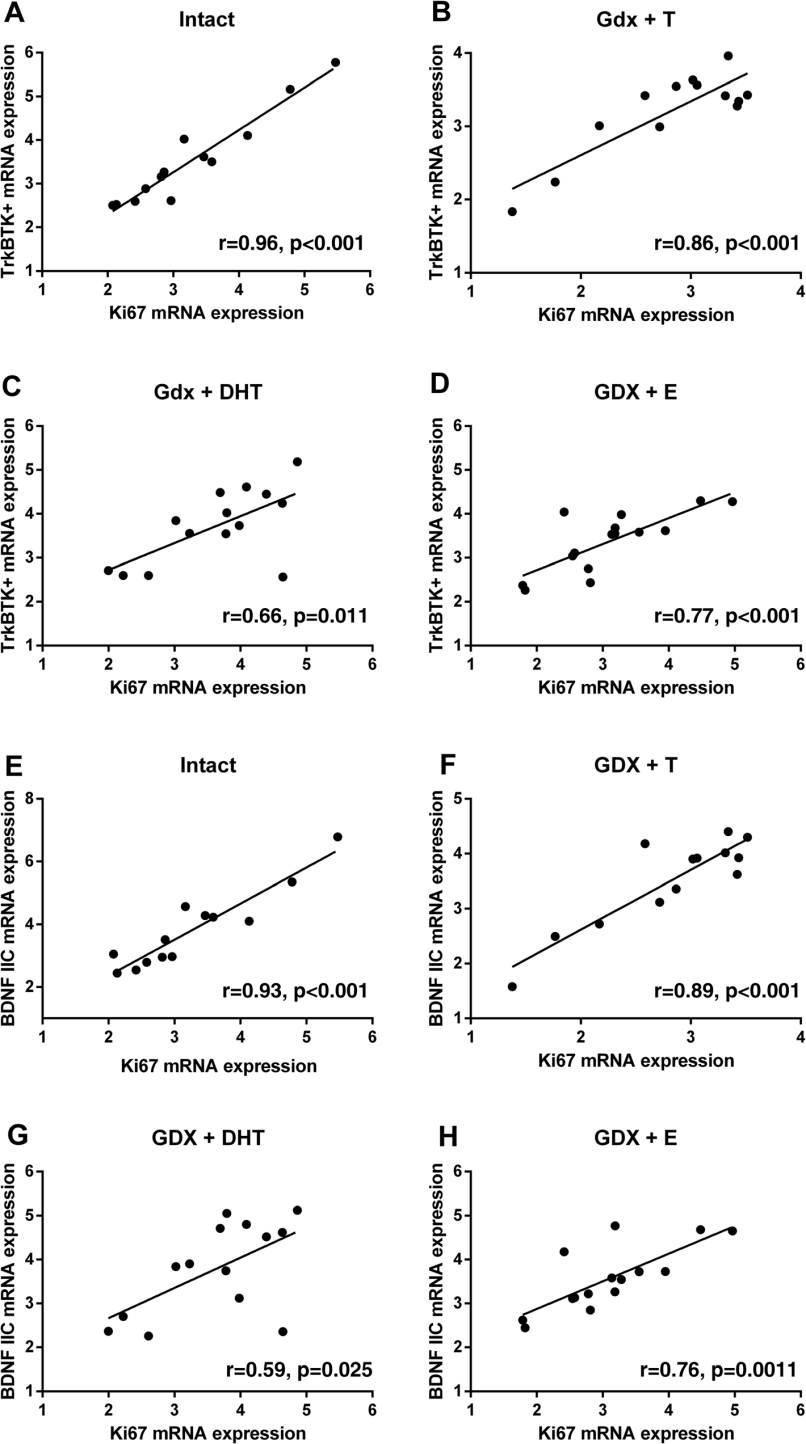
**Correlations between TrkB-TK+ and BDNF IIC mRNA expression and Ki67 mRNA expression within separate rat treatment groups.** In **(A)** Intact, **(B)** Gdx + T, **(C)** Gdx + DHT and **(D)** Gdx + E groups TrkB-TK+ and Ki67 mRNA expression were positively correlated. In **(E)** Intact, **(F)** Gdx + T, **(G)** Gdx + DHT and **(H)** Gdx + E groups BDNF IIC and Ki67 mRNA expression were positively correlated. Gdx + T = gonadectomised + testosterone replacement, Gdx + DHT = gonadectomised + dihydrotestosterone replacement, Gdx + E = gonadectomised + estradiol replacement.

In order to determine if the relationship between cell proliferation and BDNF/TrkB depended on the sex steroid environment, we separated rats into treatment groups and performed separate regression analysis within each group (Table 3). We found that Ki67 mRNA expression and TrkB-TK+ mRNA levels were only correlated when the rat was also exposed to sex steroids i.e. Intact (*r*_11_ = 0.96, *p* = 0.00000025), Gdx + T (*r*_11_ = 0.86, *p* = 0.00017), Gdx + DHT (*r*_12_ = 0.65, *p* = 0.011) and Gdx + E (*r*_13_ = 0.77, *p* = 0.00072) groups (Figure 8A-D); and that these correlations between TrkB-TK+ mRNA level and Ki67 mRNA level were absent from the Gdx group where the circulating levels of sex steroids were extremely low to non-detectable (*r*_11_ = 0.40, *p* = 0.17). We found a similar relationship between Ki67 mRNA expression and certain BDNF transcripts. BDNF I, IIC (Figure 8E-H), III and VI mRNA levels were only correlated with Ki67 mRNA in the presence of sex steroids (Table 3). BDNF IIA and BDNF IV did not show a relationship with Ki67 mRNA in any treatment group (all *p* > 0.05).

**Table 3:** **Correlations between Ki67 mRNA expression and BDNF/TrkB transcript mRNA expression within separate rat treatment groups**

	**Gdx**		**Intact**		**Gdx + T**		**Gdx + DHT**		**Gdx + E**	
	**r-value**	**p-value**	**r-value**	**p-value**	**r-value**	**p-value**	**r-value**	**p-value**	**r-value**	**p-value**
**Ki67 vs TrkB-TK+**	0.40	0.17	**0.96**	**0.00000025** ^***^	**0.86**	**0.00017** ^***^	**0.655**	**0.011** ^**^	**0.77**	**0.00072** ^***^
**Ki67 vs TrkB-T1**	−0.036	0.90	0.11	0.73	0.56	0.056	**0.72**	**0.0054** ^**^	−0.055	0.85
**Ki67 vs TrkB-T2**	−0.31	0.28	0.077	0.80	0.12	0.69	0.26	0.38	−0.098	0.73
**Ki67 vs BDNF I**	0.50	0.067	**0.79**	**0.0037** ^**^	**0.75**	**0.0046** ^***^	**0.56**	**0.047** ^*^	0.097	0.74
**Ki67 vs BDNF IIA**	−0.40	0.16	0.26	0.39	0.43	0.14	0.054	0.86	−0.039	0.89
**Ki67 vs BDNF IIC**	0.45	0.11	**0.93**	**0.000003** ^***^	**0.89**	**0.000040** ^***^	**0.59**	**0.025** ^*^	**0.76**	**0.0011** ^***^
**Ki67 vs BDNF III**	0.092	0.75	**0.79**	**0.0037** ^**^	**0.72**	**0.0052** ^**^	**0.55**	**0.041** ^*^	**0.70**	**0.0036** ^**^
**Ki67 vs BDNF IV**	0.19	0.52	0.45	0.12	0.47	0.10	0.31	0.29	0.46	0.082
**Ki67 vs BDNF VI**	−0.016	0.96	**0.59**	**0.035** ^*^	0.49	0.090	0.28	0.33	0.18	0.53

The presence of sex steroids (Intact, Gdx + T, Gdx + DHT and Gdx + E2) changed the relationship between Ki67 mRNA expression and expression of TrkB-TK+, BDNF I, BDNF IIC and BDNF III compared to Intact and Gdx + DHT groups. *p < 0.05, **p < 0.01, ***p < 0.001, bold type = p≤0.05.

## Discussion

This study sought to investigate the potential role of BDNF/TrkB signalling in the modulation of adolescent mammalian hippocampal neurogenesis by testosterone. We report that TrkB-TK- mRNA is anatomically well-positioned to mediate cell proliferation in the adult primate hippocampal SGZ, but also that TrkB-TK- appears to be robustly expressed in cells outside the SGZ. Further, TrkB-TK+ mRNA is found more widely in both the SGZ and in areas of differentiated neurons consistent with a role in maturation and survival of hippocampal neurons. While we did not find changes in the total expression of BDNF/TrkB mRNA when circulating sex steroid levels are altered in rats or monkeys, we did find that TrkB-TK+ mRNA levels, but not TrkB-TK- levels, showed a strong positive relationship with markers of cell proliferation in both species. Further, we present evidence in rats that the link between full length TrkB levels, BDNF I, IIC, III and VI levels and the level of cell proliferation markers may depend on the presence of sex steroids. Taken together with our anatomical findings above, this suggests that the regulation of BDNF signalling, potentially through full length TrkB, may indirectly mediate cell genesis in the SGZ.

We find a difference in the anatomical distribution of TrkB-TK+ vs TrkB-TK- mRNA expressing cells in the neurogenic regions of the adolescent rhesus macaque hippocampus. The highest density of TrkB-TK- mRNA expressing cells was at the border of the granule cell layer and hilus, confirming the results of a previous study in adult rhesus macaque [34]. While truncated isoforms can act to inhibit TrkB-TK+ signalling, they also have independent and unique functions (for review see [35]) and can signal through RhoGDI1 to regulate calcium influx in glia [36]. Our results would be consistent with findings suggesting that truncated TrkB is the dominant form expressed in proliferating cells and may be more salient in regulating active proliferation [18,19], as most dividing cells are found close to the granule cell layer in the SGZ of the monkey [4]. We also found a more moderate density of TrkB-TK+ mRNA+ cells in the subgranular zone, which were not identified in the previous report [34]. These TrkB-TK+ cells may represent a population of stem cells, as TrkB-TK+ has been previously identified in type-1 stem-like cells [37]. In the rat, but not in the monkey, TrkB-TK+ mRNA expression was positively associated with two markers of immature neurons (DCX and TUC4), consistent with a previous study suggesting that TrkB-TK+ is expressed in post-mitotic immature neurons (DCX+ or Tuj1+) [19,37] and primarily regulates neuronal survival and differentiation in vivo [11].

Our study, together with the previous studies mentioned above, suggest both anatomically and functionally that truncated TrkB isoforms are more poised to regulate cell proliferation in comparison to the full length form. However, we found a positive association between Ki67+ cell density in the dentate gyrus and TrkB-TK+ mRNA expression in the dentate gyrus. In the rat, hippocampal Ki67 mRNA levels were positively associated with hippocampal TrkB-TK+ mRNA levels. Ki67 did not show a relationship with levels of the truncated TrkB isoforms in either species.

Thus, we suggest that TrkB-TK+ signalling may also influence cell proliferation, possibly through an indirect route as the majority, but not all, of cells in the subgranular zone where division is taking place express TrkB-TK- not TK+. Future studies, with double-labelling techniques are required to confirm the phenotype of TrkB-TK+ cells located in the SGZ. Additionally, TrkB-TK+ is known to be expressed in stem cells in the SGZ [37], and BDNF may have a direct influence on hippocampal stem cell division and more BDNF could lead to the generation of more Ki67+ precursor cells. Alternatively, BDNF may be acting indirectly via maturing/mature hippocampal neurons that also express TrkB-TK+, and may be able to trans signal to dividing progenitors through an intermediary signalling mechanism that requires further exploration.

Contrary to our hypothesis, adolescent gonadectomy (rat and monkey) or sex hormone replacement (rat) did not alter mRNA expression of full length or truncated TrkB or any BDNF transcript (6–9 mRNAs) in hippocampal homogenate, as measured by RT-PCR. We selected transcripts that were among the most highly expressed in the primate and rat hippocampus, and would contribute significantly to overall levels of BDNF. However, as we did not measure every BDNF mRNA transcript it is possible that gonadectomy or sex steroid replacement have effects on the expression of transcripts that were not measured in the present study. In addition, we did not identify any changes in TrkB-TK+ or TrkB-TK- expression (density of mRNA+ cells or optical density) in neurogenic regions in the monkey (GCL/SGZ or hilus [4]) when comparing gonadectomised vs intact monkeys. Gonadectomy also did not appear to greatly alter the anatomical positioning of TrkB-TK+ or TrkB-TK- mRNA+ cells in the hilus vs subgranular zone (data not shown). However as only a limited amount of these regions were surveyed in our study, future studies employing different counting strategies would be required to more definitely address if gonadectomy alters the proportion of TrkB-TK+/TrkB-TK- in hippocampal subregions.

Some prior studies indicate that gonadectomy and sex steroid replacement does not alter hippocampal BDNF mRNA expression in male adult rats [38] or BDNF protein levels in adolescent mice [39] or aged male rats [40]. Hippocampal BDNF and TrkB mRNA levels may not undergo large changes from adolescence to adulthood in rats or humans [39,41,42], suggesting that TrkB and BDNF expression in the hippocampus may not be responsive to hormonal changes associated with adolescence. DHT has been shown to alter hippocampal TrkB-TK+ protein expression in male adolescent mice in a regionally specific manner, with increases in ventral hippocampus and decreases in dorsal hippocampus [39]. We did not examine dorsal/ventral (rat) or anterior/posterior regions (monkey) separately, and this may have masked any such anatomically opposing changes. Overall, in contrast to the songbird striatum [Higher vocal centre (HVC)/subventricular zone neurogenic system] [5], we suggest that testosterone influences mammalian hippocampal neurogenesis at adolescence via routes other than changes in hippocampal BDNF mRNA levels.

While we did not find a change in TrkB mRNA levels, BDNF can also signal via the low affinity receptor p75 to influence cell survival as well as apoptosis [43]. It is unclear if p75 expression in the hippocampus could be influenced by testosterone; however androgens can increase p75 expression in the spinal nucleus of the bulbocavernosus (SNB) and in the forebrain [44,45]. Testosterone has been shown to antagonise the actions of nerve-growth factor (NGF) [46] and androgens may alter NGF levels in the hippocampus [45]. TrkC and ciliary neurotrophic factor receptor (CNTFR) mediate actions of testosterone on cell survival in the SNB [47]. In the song bird HVC, the mechanism of testosterone’s action on neurogenesis also involves vascular endothelial growth factor (VEGF) [5]. Thus, testosterone may similarly have actions on mammalian hippocampal neurogenesis via modulation other growth factors or growth factor receptors, which warrant further investigation.

In rats, we found no significant effect of gonadectomy on mRNA expression of neurogenic markers (Ki67, DCX and Tuc4) in the adolescent hippocampus. Our results are consistent with a previous study in adolescent rats which found that three weeks of gonadectomy did not alter the number of immature neurons [DCX+ cells, [48]], and with our previous study that found gonadectomy did not alter cell proliferation (Ki67+ cells) in male adolescent rhesus macaques [4]. In contrast, in our previous study in the monkey, we found that markers of immature neurons (DCX mRNA expression and proportion of Prox1+/BrdU+ cells) were increased by gonadectomy. It may be difficult to compare between our studies, as our previous study in monkeys only measured the density of proliferating (Ki67+) and surviving (BrdU+), and the present study is examining Ki67 mRNA in hippocampal homogenate. However, in the monkey, immature neuronal marker (DCX) mRNA expression in hippocampal homogenate was positively correlated with measures of cell survival (BrdU+ cells), suggesting these measures may be monitoring related events. The contrast may be due to species differences in the time course of neurogenesis, and/or the timing of gonadectomy and sex hormone replacement relative to the neurogenic time course. For example in the rat, DCX is highly expressed in stages of neuronal maturation, from proliferating progenitors to cells that are 14 days old, before being downregulated [49] and we measured DCX after 2 weeks of “treatment”. Thus, DCX expressing cells would represent a mixture of cells that have proliferated before, during and after the start of the increase in natural or exogenous testosterone. In the monkey, gonadectomy was conducted over two years and neurons are thought to take up to six months to mature in this species [50], so these neurons would have proliferated and differentiated well into the period of experimental hormone manipulation. It is possible that cells that proliferate in the absence of testosterone may lack response to testosterone later in their life course, although to our knowledge this has not been investigated thus far. Although the experimental interval of sex steroid manipulation in both studies represented a similar period of time in terms of adolescent development, the study in rodents is likely examining more immediate influences on the neurogenic processes.

Testosterone can be aromatised to estradiol and thus can signal via both AR and ER pathways. Testosterone may have direct effects on neurogenesis via ERα or ERβ, which are expressed in proliferating cells (Ki67+) and immature neurons (Dcx+) in male rats [51]. In male rats, AR appears to not be expressed in immature neurons (Dcx+) [52], but may be expressed in stem cells (Nestin+) [53]. Testosterone may therefore have direct effects on stem cell proliferation via AR, but may have indirect (ER-mediated) effects on cell survival. We investigated possible independent contributions of androgenic or estrogenic mechanisms via replacement with testosterone (AR and ER signalling), 17β-estradiol (ER signalling) or dihydrotestosterone (primarily AR signalling). While gonadectomy did not alter neurogenesis compared to intact rats, testosterone replaced rats showed a trend to decreased cell proliferation compared gonadectomised rats that received no replacement. While it may be informative to measure local sex steroid levels within the hippocampus, evidence from adult rats suggest a high degree of correlation between circulating testosterone and DHT levels and levels of the same hormones within the hippocampus, and that gonadectomy greatly decreases local testosterone levels in the hippocampus [54]. This suggests that immediate and sustained supraphysiologcal levels of circulating testosterone experienced by the Gdx + T group [22], may also reflect testosterone levels in the hippocampus, and have the potential to influence neurogenesis at adolescence beyond the more gradual natural rise in testosterone experienced by intact animals from day 44 to day 60 [26]. Dose and duration of treatment dependent effects of testosterone on cell survival have been previously reported in adult male rats [2,55] and may also exist during adolescence.

Estradiol or DHT replacement did not influence expression of any neurogenic marker. Our results are consistent with previous studies suggesting that while estradiol influences neurogenesis in female rats, it does not influence cell proliferation or neuronal differentiation, at least to the same extent, in adult male rats [2,56]. Our results support the hypothesis that the level impact of estradiol in males may be consistent across adolescent to adult development. However, our study and previous studies examined effects in gonadectomised male rats, which appear to have comparable levels of estradiol in the hippocampus compared to intact animals [54]. This apparent lack of effect of estradiol replacement may be because endogenous ER mediated signalling in the hippocampus is not greatly decreased in these studies. To determine the effects of estradiol signalling on hippocampal neurogenesis in male rats, additional studies are required that specifically manipulate estrogen levels or that may block local production of estradiol within the hippocampus.

It is unknown if there are adolescent-specific changes in the influence of estradiol on neurogenesis in female rats, as there have been no studies to our knowledge on the effect of sex hormone manipulation in adolescent female rats. Similarly, DHT treatment has been previously shown to impact cell survival (not assessed in this study), but does not impact cell proliferation or differentiation in adult male rats [2,52]. As we found that testosterone, but not estradiol or DHT, putatively alters cell proliferation we suggest that combined influence of androgenic and estrogenic signalling mechanisms may be required for testosterone to exert its influence on neurogenesis during rodent adolescence.

Supraphysiolocial testosterone may also influence cell proliferation via sex steroid receptor independent mechanisms. High levels of testosterone antagonise the glucocorticoid receptor in muscle tissue [57], and decrease circulating adrenocorticotropic hormone (ACTH) levels following stress [58]. There is abundant evidence that stress can influence hippocampal neurogenesis [59,60]. Additionally, there is evidence of complex interactions between sex and stress in the regulation of post-natal hippocampal neurogenesis and male, but not female, adult rats showed decreased cell proliferation in response to acute stress [61,62] suggesting that testosterone may exacerbate acute stress effects. However, testosterone appears to buffer the effects of chronic stress, as gonadectomised male rats show exaggerated decreases in neurogenesis in response to chronic stress compared to intact animals [3]. Thus the putative effects of testosterone replacement on cell proliferation seen in our study may be due to interactions with stress signalling pathways.

**Figure 9:**
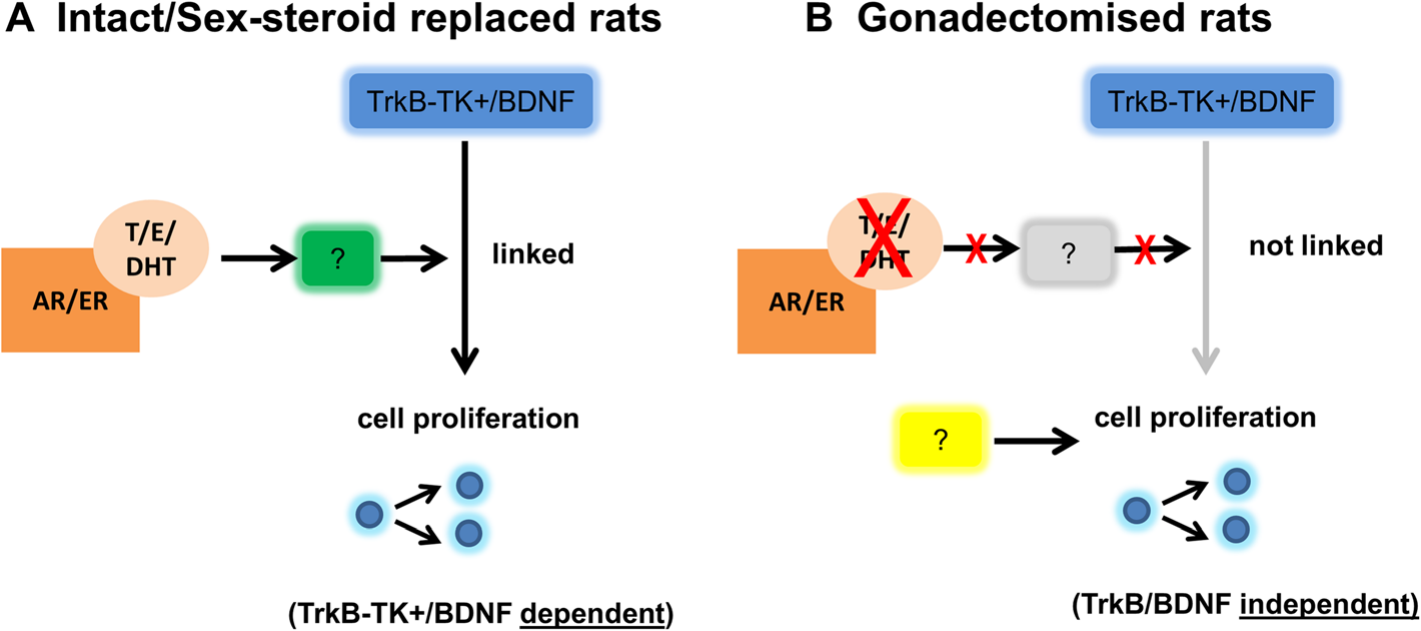
**Proposed model of cell proliferation in the presence and absence of sex steroids. (A)** AR/ER driven expression of co-factors (green square) may be required for the modulation of cell proliferation by TrkB-TK+ and BDNF. **(B)** In the absence of sex steroids, downstream co-factors are not expressed, leading to cell proliferation that is not modulated by TrkB-TK+/BDNF expression and instead by other factors (yellow square).

Although manipulation of sex steroid levels did not alter total hippocampal BDNF/TrkB mRNA levels, but as testosterone may alter cell proliferation in the rat and this was related to TrkB-TK+ expression, we sought to determine if the relationship between Ki67 and BDNF/TrkB mRNA expression would be altered in different sex steroid environments. Sex steroid removal via gonadectomy uncoupled the relationship between TrkB-TK+ and cell proliferation. Similarly, levels of several BDNF transcripts (I, IIC, III and VI) were only related to cell proliferation in the presence of sex steroids. The strongest and most numerous correlations were in the intact and in the testosterone replaced groups, suggesting this “coupling” effect may require both androgenic and estrogenic mechanisms. It is possible that sex steroids control the expression of shared factors that influence both proliferation and TrkB-TK+/BDNF expression (Figure 9).

## Conclusion

While changes in the overall levels of hippocampal BDNF/TrkB mRNA do may not mediate the influence of testosterone on neurogenesis at adolescence in the mammalian hippocampus, BDNF and TrkB-TK+ mRNA levels do appear to be indirectly related to cell proliferation (Figure 9). Our results also suggest that the relative contribution of BDNF/TrkB signalling to proliferation may change with changes in sex steroid levels during adolescence. Understanding the contribution of sex hormone and neurotrophin signalling to neurogenesis during normal adolescence may help to understand disorders that can arise at this time and involve disruption to these processes, such as schizophrenia and depression [63-66].

## Abbreviations

AR: Androgen receptor
BDNF: Brain-derived neurotrophic factor
BrdU: Bromodeoxyuridine
DCX: Doublecortin
DG: Dentate gyrus
DHT: Dihydrotestosterone
E: Estradiol
ER: Estrogen receptor
Gdx: Gonadectomy
HVC: Higher vocal centre
SGZ: Sub-granular zone of dentate gyrus
T: Testosterone
TrkB: Tropomyosin related kinase B receptor
TrkBT1/T2: Truncated TrkB receptor (rat)
TrkBTK-: Truncated TrkB receptor (monkey and human)
TrkBTK+: Full length TrkB receptor
Tuc4 (DPYSL3): TOAD [Turned On After Division]/ Ulip/CRMP (dihydropyrimidinase-like 3)
